# A cohort study for the development and validation of a reflective inventory to quantify diagnostic reasoning skills in optometry practice

**DOI:** 10.1186/s12909-022-03493-6

**Published:** 2022-07-11

**Authors:** Amanda K. Edgar, Lucinda Ainge, Simon Backhouse, James A. Armitage

**Affiliations:** School of Medicine (Optometry), Deakin University, 75 Pigdons Road, Waurn Ponds, 3216 Australia

**Keywords:** Diagnostic reasoning, Optometric education, Self-reflective inventory, Structured memory, Flexibility in thinking

## Abstract

**Background:**

Diagnostic reasoning is an essential skill for optometry practice and a vital part of the curriculum for optometry trainees but there is limited understanding of how diagnostic reasoning is performed in optometry or how this skill is best developed. A validated and reliable self-reflective inventory for diagnostic reasoning in optometry, would enable trainees and registered practitioners to benchmark their diagnostic reasoning skills, identify areas of strength and areas for improvement.

**Methods:**

A 41 item self-reflective inventory, the Diagnostic Thinking Inventory, used extensively in the medical field was adapted for use in optometry and called the Diagnostic Thinking Inventory for Optometry (DTI-O). The inventory measures two subdomains of diagnostic reasoning, flexibility in thinking and structured memory. Context based changes were made to the original inventory and assessed for face and content validity by a panel of experts. The inventory was administered to two groups, experienced (qualified) optometrists and second-year optometry students to establish validity and reliability of the self-reflective tool in optometry.

**Results:**

Exploratory Factor Analysis uncovered 13 domain specific items were measuring a single construct, diagnostic reasoning. One misfitting item was removed following Rasch analysis. Two unidimensional subdomains were confirmed in the remaining 12 items: Flexibility in Thinking (χ2 = 12.98, *P* = 0.37) and Structured Memory (χ2 = 8.74, *P* = 0.72). The ‘Diagnostic Thinking Inventory for Optometry Short’ (DTI-OS) tool was formed from these items with the total and subdomain scores exhibiting strong internal reliability; Total score C_α_ = 0.92. External reliability was established by test-retest methodology (ICC 0.92, 95% CI 0.83–0.96, *P* < .001) and stacked Rasch analysis (one-way ANOVA, F = 0.07, *P* = 0.80). Qualified optometrists scored significantly higher (*P* < .001) than students, demonstrating construct validity.

**Conclusion:**

This study showed that the DTI-O and DTI-OS are valid and reliable self-reflective inventories to quantify diagnostic reasoning ability in optometry. With no other validated tool to measure this metacognitive skill underpinning diagnostic reasoning a self-reflective inventory could support the development of diagnostic reasoning in practitioners and guide curriculum design in optometry education.

**Supplementary Information:**

The online version contains supplementary material available at 10.1186/s12909-022-03493-6.

## Background

Diagnostic reasoning is an essential competency skill for optometrists as it underpins professional judgement, allowing for optometry practitioners to best use evidence-based practice and provide quality patient care [1, 2]. There is no general definition of diagnostic reasoning though theoretical explorations of this cognitive action discuss a multidimensional complex process [3]. For example the ‘dual-processing theory’ details the important involvement of analytical and non-analytical thinking in diagnostic reasoning [3]. Across professions there continues to be a concerted effort to define and benchmark diagnostic reasoning and variations in research findings pose a challenge for the teaching and evaluation of this required skill that is yet to be fully explored in optometry.

There is no generally accepted definition of diagnostic reasoning in optometry however optometrists, like other health professionals, use diagnostic reasoning in clinical practice. Diagnostic reasoning is a requirement for optometric practice, with the Optometry Australia Entry Level Competency Standards outlining that a novice practitioner is expected to integrate clinical expertise, patient preferences and evidence based-practice when making clinical decisions [2, 4]. Faucher et al.*,* in a study that investigated diagnostic reasoning used by optometrists, demonstrated qualified optometrists do utilize both analytical and non-analytical modes of diagnostic reasoning concurrently when performing an eye examination [5]. This study describes diagnostic reasoning as found by Faucher et al.: Diagnostic reasoning is applied by an optometrist during the initial interviewing of a patient, through planning the remainder of the consultation, which includes selecting appropriate tests to investigate their hypothesis and concurrently analyzing test results to refine their mental representation of the patients problem [5]. This contains similarities to the integrated model of clinical reasoning described in medical practice [6]. The role of an optometrist, however, differs from that seen in nursing practice or general medical practice due to the requirement to plan, undertake and analyze diagnostic tests to implement management plans in the absence of a clinical team and to review these decisions. In comparison to general medical practitioners who benefit from the input of other specialties to routine diagnostic testing such as pathologists or radiologists, an optometrist is potentially more open to error as they may have sole responsibility for the application and interpretation of most diagnostics. It is therefore feasible that there are unique diagnostic reasoning steps that are crucial to the development of professional competence as an optometrist. There is thus a need to develop a level of self-reflection through training, yet a paucity of research on diagnostic reasoning in optometrists leads to educators relying on the conflation of evidence from other health professions to teach this skill.

An added benefit to optometry education would be to incorporate a validated and reliable tool to measure the way optometrists conduct diagnostic reasoning, for use when specifically evaluating diagnostic reasoning or to design diagnostic reasoning learning activities. Bordage et al.*,* [7] developed the Diagnostic Thinking Inventory (DTI) to quantify diagnostic thinking in medical students and physicians with greater than 3 years’ experience. The tool consists of 41-items designed to measure self-assessed diagnostic reasoning ability and the two subdomains of diagnostic reasoning, flexibility in thinking (the extent that processes can be applied during diagnostic reasoning) and structured memory (knowledge stored and readily available during the diagnostic process) [7]. These two subdomains have previously been identified to be performed more effectively by experienced optometrists as they are readily able to access a more structured knowledge base and synthesize important data to construct a mental representation of the patient [5]. Validation of the DTI in optometry would be beneficial given that the DTI has been widely used to assess and monitor diagnostic reasoning development, to evaluate diagnostic reasoning teaching methods and has been successfully adapted to other health professions [8–15].

A Diagnostic Thinking Inventory for Optometry (DTI-O) has potential to help monitor the development of diagnostic reasoning ability for practicing clinicians and students. As a reflective tool, the structure of the DTI and its subdomains are consistent with how an optometrist’s diagnostic reasoning ability differs with experience. It is broad enough to be easily understood without expertise in diagnostic reasoning theory and could therefore be used to support self-regulated learning and student agency, and it’s subdomains are consistent with how optometrists diagnostic reasoning ability differs with experience [5]. Educators require a robust evaluative tool in order to develop, refine and validate the effectiveness of scholarly based teaching interventions or learning tools it is important for us to use a robust evaluative tool. A second consideration is that the DTI-O may have the greatest utility as a tool to track development of diagnostic reasoning and therefore must be taken on multiple occasions. In this instance, completing the 41-item inventory on several occasions might represent a barrier to use given the length of time taken to complete. Defining the minimum number of items required may shorten the inventory and increase the utility of the tool. The purpose of this cross-sectional cohort study is to uncover the underlying structure of a relatively large set of items using Rasch analysis and Exploratory Factor Analysis [16]. This process aims to identify key items and determine the reliability and validity of the resulting DTI-O through comparing results between experts and novices. A valid inventory is hypothesized to produce significantly lower diagnostic reasoning scores for students compared to those of qualified optometrists.

## Methods

### Participants and setting

Participants were recruited for this cross-sectional cohort study and assigned to one of two groups, an expert group and a novice group. The novice group consisted of second-year optometry students enrolled in the Bachelor of Vision Science/Masters of Optometry at Deakin University and the expert group were qualified optometrists. This research was reviewed by an independent ethical review board and conforms with the principles and applicable guidelines for the protection of human subjects in biomedical research. This research follows the tenets of the Declaration of Helsinki [17]. After an explanation of the study was provided, written informed consent was obtained from all participants.

All 81 students enrolled in their second-year were invited to participate in the study. The Deakin University optometry program teaches across three trimesters per year, allowing the course to be taught in three and a half years rather than five. Second-year is a preclinical course stage where students are taught through a problem-based learning curriculum and therefore have limited exposure through coursework to hypothetico-deductive diagnostic reasoning. A convenience sampling method was used to recruit qualified optometrists through professional development seminars and alumni networks.

Participants from the novice group were excluded if they were repeating any second-year optometry units, had studied optometry previously or studied another health-related field in higher education to limit the variability of previous exposure to diagnostic reasoning. Participants from the expert group were excluded if they were a recent graduate (less than six months from graduation) or had less than three years’ experience as a qualified optometrist as this period of professional life is often characterized by rapid changes in clinical exposure, reasoning skills and confidence.

### Materials

The DTI-O (Additional file 1) was adapted from the DTI developed by Bordage et al.*,* that reported acceptable reliability (Cronbach’s alpha = 0.83) in a validation study [7]. The DTI-O, like the original inventory developed by Bordage et al.*,* contains 41-items [7]. These items align to one of the two subdomains, flexibility in thinking (21 items) or structured memory (20 items). To adapt to the profession of optometry as it is practiced in Australia, context-based changes were made to 6 items after an expert panel review to ensure face validity. In addition, demographic questions for age, gender, education level, scope of practice and experience supervising optometry students were added to the DTI-O for this study. These alterations did not change the original structure or order of the items.

Each item consisted of a stem followed by two statements which are the opposite of each continuum on a 6-point semantic differential scale. The items, as per the original DTI, alternate positive statements from the right and left-hand side of the page to avoid complacency. The score is added (with appropriate correction of reverse scored items), so a higher score represents more advanced diagnostic reasoning with a maximum of 246 for the total score, 126 for flexibility in thinking and 120 for structured memory as per the original DTI. It was acknowledged that participants may agree with both statements in different situations and contexts. For each item, participants decided where on the continuum they sit most of the time. For this study, qualified optometrists were asked to reflect on their experience in general optometry practice and students on their experience in case-based learning. To confirm face and content validity, the inventory it was piloted by a panel of experts.

### Procedures

The 41-item inventory was administered as a paper-based anonymous inventory. Participants from both groups completed the inventory to assess construct validity based on the hypothesis that qualified optometrists would have more highly developed diagnostic reasoning skills and score higher in comparison to undergraduate students. The expert group was instructed to complete a second attempt of the same 41-items after a 3-week time gap to assess the external reliability of the DTI-O in a test-retest setting [18]. This timeframe was selected as it has been suggested that between two to four weeks is long enough to ensure there is no false agreement due to remembrance and short enough so there are no false results from learning between test timepoints [18].

Scores on the DTI-O were recorded electronically and input into IBM SPSS Version 25 for statistical analysis.

### Statistical analysis

Quantitative analysis was performed on the results of the DTI-O. Construct validity was determined with Rasch Measurement Theory, Exploratory Factor Analysis and comparison of the two groups’ quantitative results from the inventory and its two subdomains. A *P* value less than .05 was statistically significant.

#### Rasch measurement theory - Rasch analysis

The full 41 item DTI-O survey results were initially analyzed using the Rasch measurement model for polytomous responses to investigate the underlying latent trait of the item difficulties and person abilities [19, 20]. Rasch analyses were carried out using the RUMM2030 software package [21]. The decision as to which parameterization of the polytomous model to use (unrestricted partial credit or rating scale), was made after examining the outcome of Fisher’s likelihood ratio test [21]. The assumption of unidimensionality in the items, in both the full survey results and the SM and FT subdomains, was examined by comparing the person estimates from two item subscales based on the patterning of item loadings on the first residual factor [22]. Exploratory Factor Analysis and reduction of the number of items in the survey was then performed, as detailed below, and Rasch analysis of the resulting reduced-item survey was performed to validate the selected questions. The principles of analysis and reporting outlined by Tennant and Conaghan were followed when examining and validating the shortened survey [23]. In RUMM2030, item fit residuals falling within the − 2.5 to + 2.5 range are considered to fit the model and this criterion was used as a first-pass analysis of item fit [21]. All items, in particular items showing fit residuals outside this range or with significant chi-square statistics, were evaluated for retention or removal from the reduced-item survey using all available fit statistics [21].

#### Exploratory factor analysis

Scores on the DTI-O were recorded electronically and input into IBM SPSS Version 25 for Exploratory Factor Analysis. Exploratory Factor Analysis was used to establish if all domain specific items (flexibility in thinking and structured memory) that were predesignated by previous research in the medical field primarily assessed one construct, diagnostic reasoning in optometry, after checking if the data were normally distributed [24]. Using this inventory in a next context, optometry, Exploratory Factor Analysis was used to identify any unrelated items that should be removed based on cut-off points in existing literature [24]. Prior to undertaking the analysis adequacy of the sample size was assessed with the following criteria: Kaiser-Myer-Olkin sampling adequacy was met with a value above 0.6; Kaiser’s criterion of 1 for eigenvalues; there were no outliers; goodness of fit was assessed with Bartlett’s test of sphericity with 0.5 as the cut-off point [25].

Principle axis factor analysis was performed with oblique rotation (Promax) as we anticipated that factors would be corelated as the entire inventory aimed at practitioners self-reflection on diagnostic reasoning [25]. Principle axis factor analysis would identify the lowest number of factors that describe common variance in the variables [25]. Factors were retained if they had eigenvalue’s greater than 1 and using descending variances from a scree plot [25]. In accordance to literature factors were removed if they had less than 5 items with a factor loading above 0.5 [26]. Items were also removed, if commonalities were below 0.3, or if they had similar factor loadings on several factors or cross loaded on more than one factor with factor loadings equal to or above 0.32 [26, 27]. Internal consistency of each factor was assessed with Cronbach’s α for reliability, with a value of α > 0.70 considered to have sufficient inter-item consistency based on literature [28]. After deletion of an item or factor the analysis was repeated.

For reliability, internal consistency and external reliability were both assessed. Internal consistency was tested with Cronbach’s alpha with a value greater than 0.7 considered to be sufficient. External reliability was assessed using test-retesting statistical comparison (repeatability and agreement) of the results from the expert group’s first and second attempts using intraclass correlation coefficients (ICC) [26]. The participants with less than three years’ experience as a qualified optometrist were not included in the analysis of external reliability given the likelihood that results may be affected by the rapid nature of their learning progression and diagnostic reasoning development.

## Results

### Descriptive statistics

71 participants and 98 inventories were included in the analysis. For the novice group 39 second year optometry students (response rate 49%) consented to participating in the study and the majority were female (*n* = 21), which is representative of the cohort.

The expert group consisted of 32 qualified optometrists, 27 returned a second attempt at the inventory, and the majority were female (*n* = 22). All qualified optometrists that participated reported that prescribing topical ocular medications was within their scope of practice. Of these participants 60% had experience as clinical supervisors of optometry students, 34% had a Bachelor’s Degree, 53% a Master’s Degree and 13% had a PhD. It should be noted that due to progressive changes in the entry level training for Australian optometrists over the last two decades the entry level qualification for the profession was at the Bachelor’s level until approximately 2010 therefore practitioners with more clinical experience in this sample would likely have Bachelors qualification and more recent graduates the Masters level qualification.

### Validity

Face and content validity were assessed by a panel of experts, two community optometrists and three academics who have contemporary clinical practice and a research interest in diagnostic reasoning. They were provided draft items from the DTI and asked to comment on face and content validity by reviewing the clarity of items and relevance to optometry ensuring that the inventory and the subdomains represented aspects of diagnostic reasoning applicable to an optometry setting. The inventory was modified based on this review, with minor context-based changes to 6 items to allow applicability to optometry. For example, ‘laboratory tests’ were changed to ‘clinical tests’. These alterations did not alter the original structure of the items or the meaning. There was unanimous agreement from the panel of experts that the inventory is a self-assessment tool to measure diagnostic reasoning in optometry.

#### DTI-O Rasch measurement theory

##### DTI-O Rasch analysis – test of fit

An initial examination of the data using all 41 questions was undertaken using Rasch analysis. The repeated measure data from the experts at time points 1 and 2 were both included in the analysis by stacking the data to allow for assessment of repeatability of the survey [26]. A significant result in the likelihood ratio test (*P* < 0.05) indicated that the polytomous data should be analyzed using the unrestricted partial credit model [21]. The total chi-square item-trait interaction statistics (χ^2^ = 291.99, df = 82, *P* < 0.0001) indicated a poor fit of the data to the model. Item fit residuals were close to the expected values (0.38 ± 1.37) but person fit residuals (− 0.24 ± 2.28) were outside expectations, further highlighting misfit with the model. The person separation index was 0.91, indicating a high ability to detect misfit within the model. There were no extreme values present within the data, suggesting no ceiling or floor effects were present [29].

To explore the misfit in the model, assuming a violation of unidimensionality within the survey existed, a principal component analysis was undertaken. Principal component 1 (PC1) had an Eigenvalue of 4.86, which accounted for 11.85% of the total variance within the model, which is greater than would be expected from a unidimensional instrument [30]. The items were divided into two subscales based on positive and negative loadings on PC1 and the resulting independent t-tests were examined [22]. Overall, 38 participants (38.78%) showed significantly different person estimates between the two subscales at the *P* = 0.05 level, with 17 (17.35%) continuing to show differences in their person estimates at the *P* = 0.01 level. The difference in person estimates between the two subscales indicates that there is likely multidimensionality within the instrument.

##### DTI-O subdomain Rasch analysis – test of fit

As the original DTI survey was built around two subdomains (20 SM items and 21 FT items), Rasch analyses were carried out on each subdomain individually using the unrestricted partial credit model. The total chi-square item-trait interaction statistics remained poor when both the SM (χ^2^ = 117.65, df = 40, *P* < 0.0001) and FT (χ^2^ = 99.41, df = 42, *P* < 0.0001) subdomains were analyzed, indicating continued poor fit of the data to the model. Principal component analysis revealed multidimensionality was potentially still present within the SM (PC1 Eigenvalue 3.15, accounting for 15.77% of the variance) and FT (PC1 Eigenvalue 2.92, accounting for 13.89% of the variance) subdomains. Subscale analysis of the positive and negative PC1 loadings via independent t-tests showed significant multidimensionality still existed within both the SM (23 participants [23.47%] at the *P* = 0.05 level) and FT (15 participants [15.31%] at the *P* = 0.05 level) subdomains. To further reduce the subdomains and make them unidimensional, an exploratory factor analysis was undertaken.

##### DTI-O exploratory factor analysis - item reduction

**Fig. 1:**
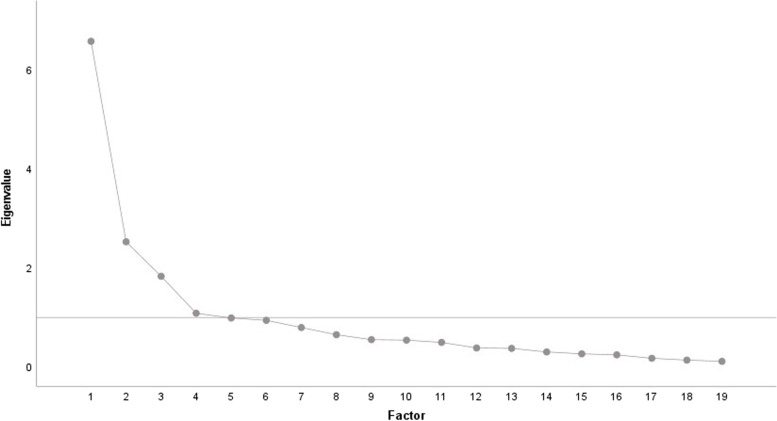
Scree plot of exploratory factor analysis. The scree plot shows the first 4 factors have an eiganvalue greater than 1

For construct validity, Exploratory Factor Analysis was performed on all results (*n* = 98) and the sample size was determined to be acceptable to perform this type of analysis with a Kaiser-Myer-Olkin value of 0.78, Bartletts’ Test of Sphericity was statistically significant, χ^2^(171) = 604.13, *P* < 0.001, and all item commonalities were above 0.8. A scree plot (Fig. 1) characterizes the relationship between the factors. Factor correlations were Exploratory Factor Analysis resulted in one major factor to be retained as it has an Eigenvalue greater than 1, with removal of 3 other factors with Eigenvalue’s greater than 1 that had factors with more than 5 items with a factor loading of 0.5 or above (Table 1). This factor had an eigenvalue of 7.86 that accounted for 34.57% of the cumulative variance of the inventory (Fig. 1). Once item reduction was completed results indicate that each of the subdomains were measuring one primary construct and the DTI-OS inventory was now a unidimensional scale with 13 items retained and the internal consistency was Cronbach α = 0.87.

**Table 1:** Factor analysis of the DTI-O results

Item number	Subdomain	Factor 1	Factor 2	Factor 3
31	SM	0.753		
25	FT	0.738		
8	SM	0.734		
27	FT	0.73		
41	FT	0.668		
5	FT	0.588	0.336	
33	SM	0.574		
9	SM	0.567		
19	SM	0.559		
4	FT	0.543		
13	SM	0.386	0.479	
15	FT	0.406	0.338	−0.5
38	FT	0.393		0.476

Principal Axis Factoring extraction method. Promax rotation method with Kaiser normalisation coverage in 6 iterations. Following convention, factors with less than 5 items with factor loadings above 0.5 were removed. Items with loadings greater than 0.3 were included

Abbreviations: *FT* flexibility in thinking, *SM* structured memory

#### DTI-OS Rasch analysis – test of fit

Following the Exploratory Factor Analysis, a 13-item shortened version of the DTI-O survey was proposed (Diagnostic Thinking Inventory for Optometry Short; DTI-OS). Rasch analysis of the DTI-OS showed better fit to the model than the full 41 item DTI-O, although the total chi-square item-trait interaction statistics continued to show a significant chi-square probability (χ^2^ = 45.29, df = 26, *P* = 0.01). Item (0.18 ± 1.62) and person (− 0.26 ± 1.46) fit residuals were close to expected values. The person separation index remained high (0.90). Principal component Eigenvalues were more evenly distributed across all principal components than in the DTI-O, although PC1 had an Eigenvalue of 1.56, accounting for 14.28% of the variance in the model. As the DTI survey was originally designed to assess two subdomains, detailed Rasch analyses were conducted on each of the DTI-OS subdomains independently to assess appropriateness of the selected items.

#### DTI-OS FT subdomain analysis – test of fit

**Fig. 2:**
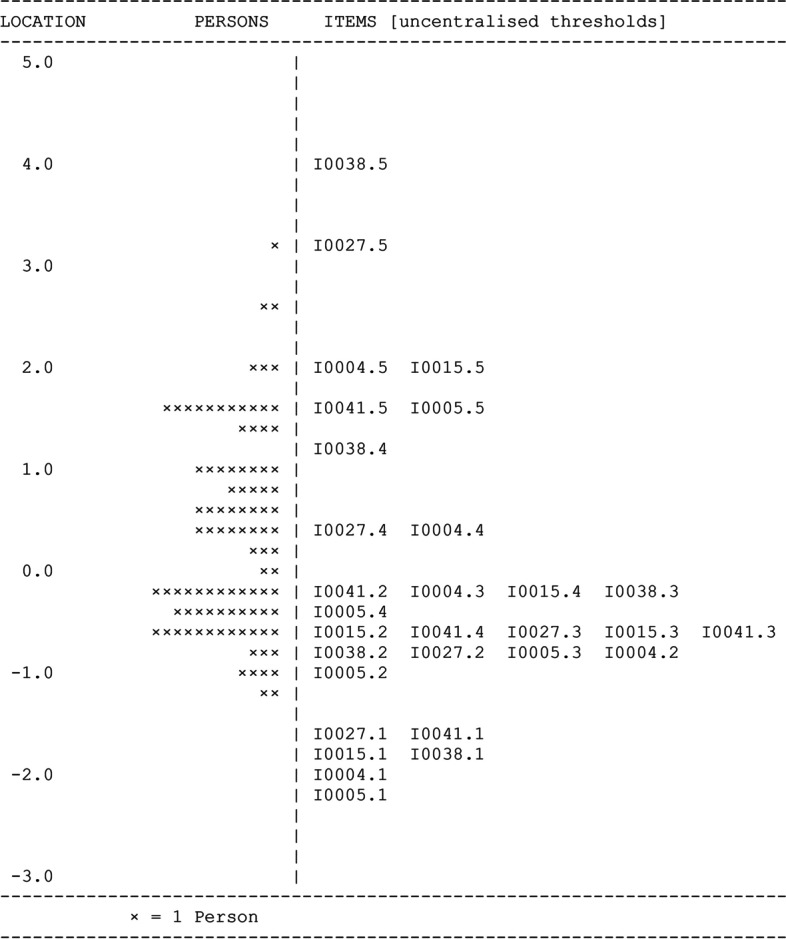
Wright map showing person and item uncentralized threshold locations for the six items in the DTI-OS FT subdomain

The six-item FT subdomain in the DTI-OS showed good fit to the Rasch model, with good chi-square item-trait interaction statistics (χ^2^ = 12.98, df = 12, *P* = 0.37). The item (0.18 ± 1.29) and person (0.28 ± 1.04) fit residuals also had close fits with expected values (Fig. 2). The person separation index was a little lower than in the full DTI-OS at 0.77, but the power of the analysis of fit was still good. The principal component values were equally distributed, which along with the non-significant chi-square statistic indicated the reduced FT items were assessing a single trait.

##### Item fit

Individual item fit residuals were checked, with no items showing fit residuals outside the expected ±2.5 logit range. None of the items had significant chi-square probabilities, indicating all items showed good fit to the model (Table 2).

**Table 2:** Individual item Rasch fit analyses for the six FT subdomain questions in the DTI-OS

Item	Location	Standard Error	Fit Residual	Chi-square	P
4	0.006	0.102	0.020	2.378	0.30
5	−0.476	0.107	−0.583	0.764	0.68
15	−0.111	0.101	2.292	4.749	0.09
27	0.189	0.107	−1.561	4.545	0.10
38	0.588	0.113	0.630	0.397	0.82
41	−0.194	0.095	0.271	0.149	0.93

Abbreviations: *FT* flexibility in thinking, *DTI-OS* Diagnostic Thinking Inventory for Optometry Short

##### Disordered thresholds and differential item functioning

Disordered thresholds were observed in item 41, with all other items showing good threshold distributions. Examination of the category response frequencies showed that there were insufficient responses in the impacted categories to indicate whether true disordered thresholds were present. Rescoring of item 41 to collapse the disordered categories was therefore not warranted in the final survey with the present data. An analysis of differential item functioning (DIF) between experts and novices revealed no significant DIF for any item.

##### Local response dependency

Person-item residual correlations were examined to look for any local response dependencies following the polytomous item dependency protocol proposed by Andrich et al. [31]. Item 38 showed a significant residual correlation (− 0.354) with item 15, and item 41 showed a significant residual (− 0.314) with item 38. All attempts to split the items to statistically determine the level of dependency present were unsuccessful due to the presence of extreme values. A thematic analysis was undertaken, with all questions deemed to be addressing different aspects of FT. It was decided to retain all questions in the FT subdomain in the final DTI-OS survey due to the good overall fit of the FT subdomain to the model.

#### DTI-OS SM subdomain analysis – test of fit

The seven-item SM subdomain in the DTI-OS showed good fit to the Rasch model, with good chi-square item-trait interaction statistics (χ^2^ = 19.56, df = 14, *P* = 0.145). Item (0.27 ± 1.31) and person (0.27 ± 1.13) fit residuals closely matched expected values. The person separation index was 0.82, and the power of the analysis of fit was good. Relatively equal distribution of principal component values and the good chi-square statistic indicated the reduced SM items were all assessing a single trait.

##### Item fit

Individual item fit residuals were checked, with item 25 showing a fit residual greater than the expected 2.5 logit threshold (3.03 logits) and a significant chi-square statistic with Bonferroni correction at the *P* = 0.05 level (χ^2^ = 10.38, *P* = 0.0056). Examination of the item characteristic curves for item 25 confirmed that it under-discriminated. No other items showed significant deviations from the expected pattern.

##### Disordered thresholds and differential item functioning

Disordered thresholds were observed in item 9, but an analysis of the category response frequencies revealed a small number of responses in many categories. There was insufficient evidence in the data to indicate true threshold disorder, and the item was not rescored in the final survey. No significant DIF between experts and novices was observed for any item.

##### Local response dependency

Item 25 showed a significant residual correlation (− 0.429) with item 8, and item 33 showed a significant residual correlation (− 0.378) with item 9. Quantifying the dependency of item 25 on item 8 revealed an estimate of the magnitude of dependence ($\hat{d}$) of 0.9955 and a variance of the mean of the estimates of dependence (${\hat{\sigma}}_d$) of 0.082 [29]. A z-score of 12.09 was calculated, which showed significant local dependence for responses to item 25 on the responses to item 8. Thematic assessment found both items addressed very similar concepts. Due to the local response dependency and poor item fit of item 25 it was decided to remove this item from the survey and repeat the Rasch analysis on a 12-item DTI-OS.

##### DTI-OS SM subdomain analysis – test of fit following item 25 removal

**Fig. 3:**
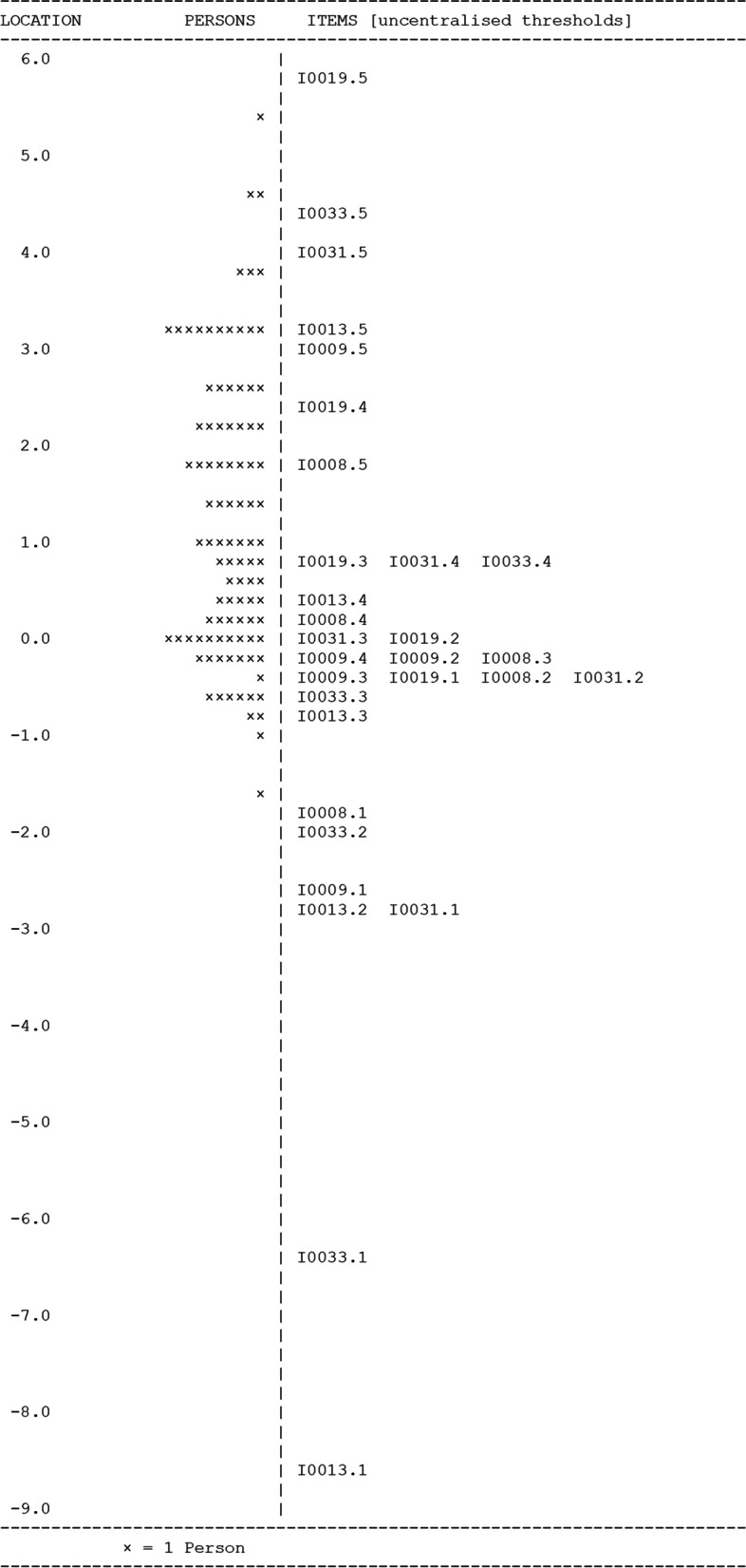
Wright map showing person and item uncentralized threshold locations for the six items in the DTI-OS SM subdomain

Removal of item 25 from the SM subdomain had minimal impact on the overall fit to the Rasch model (χ^2^ = 8.74, df = 12, *P* = 0.72; item fit residual 0.09 ± 0.48; person fit residual − 0.32 ± 1.08; person separation index 0.84) (Fig. 3). All items showed good individual fit residuals within the expected ±2.5 logit range, with no significant chi-square probabilities (Table 3). No DIF was observed between novice and expert responses for any items. Item 33 continued to show local response dependency on item 9 (0.431). The lowest category in item 33 had no responses, so it was not possible to accurately determine the magnitude of response dependency between these two items. Following thematic review of the items it was determined that they were both assessing different aspects of the SM subdomain and were therefore both retained in the final survey.

**Table 3:** Individual item Rasch fit analyses for the six SM subdomain questions in the DTI-OS

Item	Location	Standard Error	Fit Residual	Chi-square	P
8	0.037	0.111	−0.584	4.903	0.09
9	−0.042	0.121	0.375	0.478	0.79
13	−1.618	0.148	0.664	0.379	0.83
19	1.800	0.117	−0.381	2.284	0.32
31	0.443	0.122	0.180	0.225	0.89
33	−0.620	0.150	0.305	0.474	0.79

Abbreviations: *SM* structured memory, *DTI-OS* Diagnostic Thinking Inventory for Optometry Short

#### DTI-OS 12-item analysis

##### DTI-OS 12-item Rasch analysis – test of fit

Following removal of item 25 the total chi-square item-trait interaction showed a significant chi-square probability for the full 12-item DTI-OS (χ^2^ = 44.42, df = 24, *P* = 0.007). Item (0.10 ± 1.48) and person (− 0.31 ± 1.40) fit residuals remained close to expected values, with the person separation index remaining high (0.90). Principal component Eigenvalues were still unbalanced, with PC1 having an Eigenvalue of 1.79 accounting for 14.95% of the variance in the model, suggesting the two subdomains were still represented in the 12-item survey. As with the full 41-item analysis, the 12 DTI-OS items were divided into two subscales based on positive and negative loadings on PC1 [22]. A total of 10 participants (10.20%) showed significantly different person estimates between the two subscales at the *P* = 0.05 level, while 4 (4.08%) continued to show differences at the *P* = 0.01 level. The difference in person estimates between the two subscales indicates that a degree of multidimensionality likely still exists within the 12-item DTI-OS instrument, albeit less than that observed with the full 41-item survey.

##### DTI-OS 12-item analysis – item targeting

**Fig. 4:**
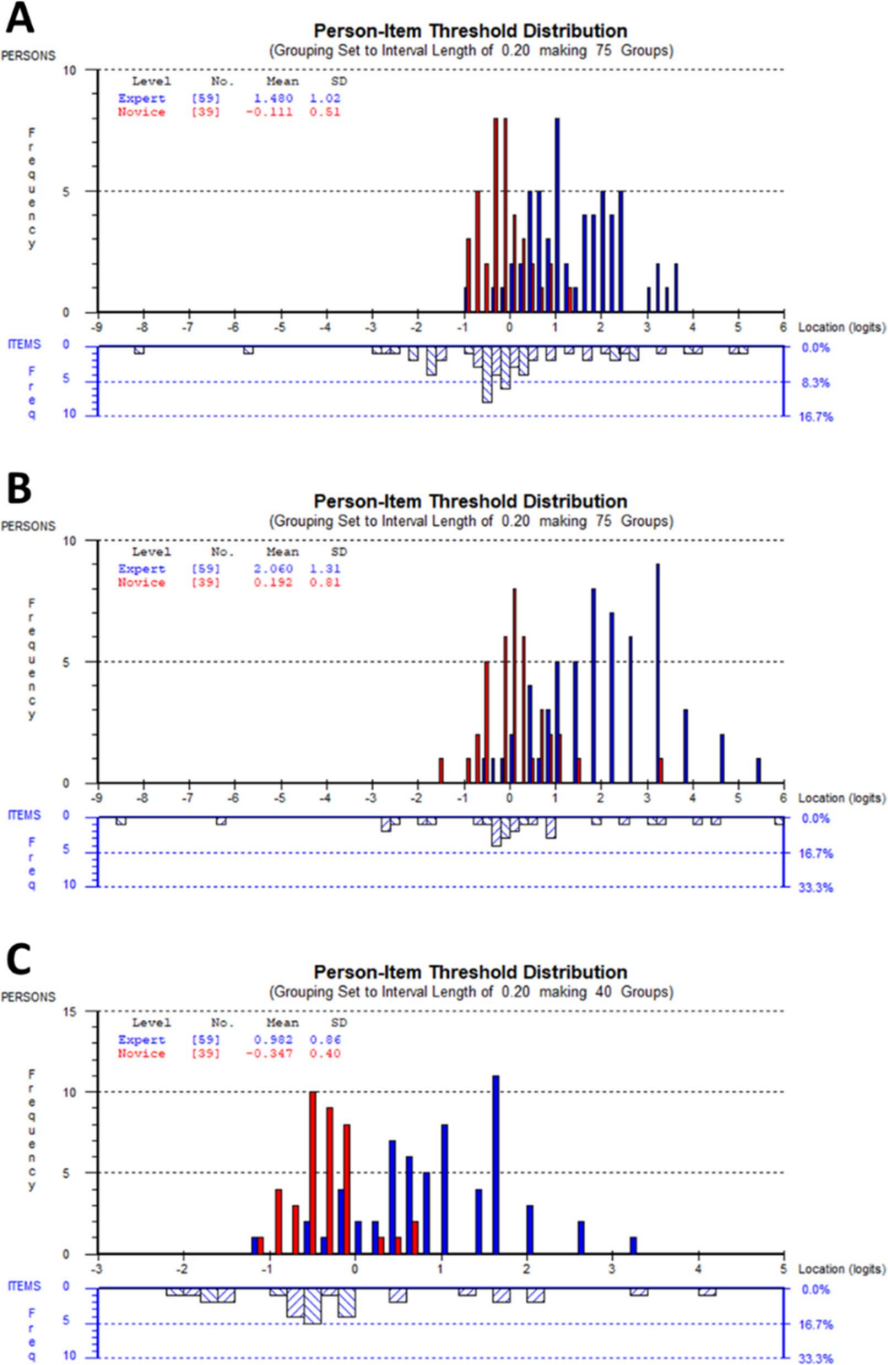
Person-item threshold distributions showing targeting of item difficulties to person abilities, in logits, for: **A** the 12-item DTI-OS survey; **B** the six SM subdomain questions in the DTI-OS; and **C** the six FT subdomain questions in the DTI-OS. The difference in mean person abilities between experts and novices was significant for all three analyses (one-way ANOVA, *P* < 0.0001 in all instances). Abbreviations: DTI-OS, Diagnostic Thinking Inventory for Optometry Short; FT, flexibility in thinking; SM, structured memory

Examining the 12-item DTI-OS, with six items in the SM subdomain and six items in the FT subdomain, revealed good targeting of the item difficulties (0.00 ± 0.82 logits) to the person abilities (0.85 ± 1.16 logits) within the survey. The mean ability of the experts (1.48 ± 1.02 logits) was significantly greater than the abilities of the novices (− 0.11 ± 0.51 logits) (Fig. 4a; one-way ANOVA, F = 80.86, *P* < 0.0001). No differential item functioning was noted for any item, indicating the discrimination in abilities between the two groups of participants was not impacted by poor item performance. Targeting of item difficulties and person abilities was not as good for the SM subdomain (0.00 ± 1.14 and 1.32 ± 1.46 logits respectively; Fig. 4b) as it was for the FT subdomain (0.00 ± 0.36 and 0.45 ± 0.96 logits respectively; Fig. 4c). The expert abilities were significantly greater than novice abilities in both the SM (2.06 ± 1.31 versus 0.19 ± 0.81 logits; F = 63.57, *P* < 0.0001) and FT (0.98 ± 0.86 versus − 0.35 ± 0.40 logits; F = 81.94, *P* < 0.0001) subdomains. The greater expert ability in the SM subdomain likely accounts for the poorer overall item targeting observed with these questions.

### Classical test theory – construct validity

**Fig. 5:**
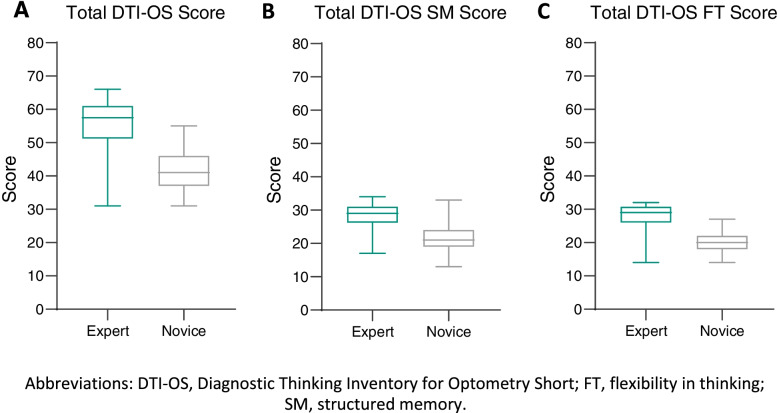
The distribution of scores for both groups; **A** DTI-OS total scores; **B** DTI-OS flexibility in thinking scores; **C.** DTI-OS structured memory scores

Further construct validity was investigated on the remaining 12 items using an independent *t*-test to determine the significant difference between the scores of both groups. The difference between scores on the DTI-O and the shortened version DTI-OS are shown in Table 4 with the mean and standard deviation of total scores, flexibility in thinking and structured memory for each administration. The results show the difference between the two groups is statistically significant with the novice group scoring lower than the group of experts with the distribution described in Fig. 5.

**Table 4:** Difference between groups

Category	Maximum possible score	Means (± SDs)		Mean difference (upper and lower bounds, 95% CI)	Independent sample ***t***-test t(df)
		Experts (***n*** = 32)	Novice (***n*** = 39)		
Total DTI-OS	72	55.88(**±** 6.89)	41.51 (**± 6.34**)	14.36 (11.22–17.50)	9.12 (69)*
SM DTI-OS	36	28.43 (**± 3.67**)	21.51 (**± 4.01**)	6.92 (5.08–8.76)	7.52 (69)*
FT DTI-OS	36	27.44 (**±** 3.67)	20.00 (**±** 3.09)	7.43 (5.72–9.15)	8.64 (69)*
Total DTI-O	246	182.34 (**±** 20.39)	155.62 (**±** 12.70)	26.72 (18.82–34.63)	6.75(69)*
SM DTI-O	120	90.75 (**±** 9.22)	76.45 (**±** 7.94)	10.90 (7.64–14.15)	6.71(69)*
FT DTI-O	126	91.59 (**±** 12.37)	79.13 (**±** 7.18)	7.50 (3.62–11.37)	4.45(69)*

*Significant at *P* < .001

Abbreviations: *DTI-O* Diagnostic Thinking Inventory for Optometry, *FT* flexibility in thinking, *SM* structured memory, *DTI-OS* Diagnostic Thinking Inventory for Optometry Short

**Fig. 6:**
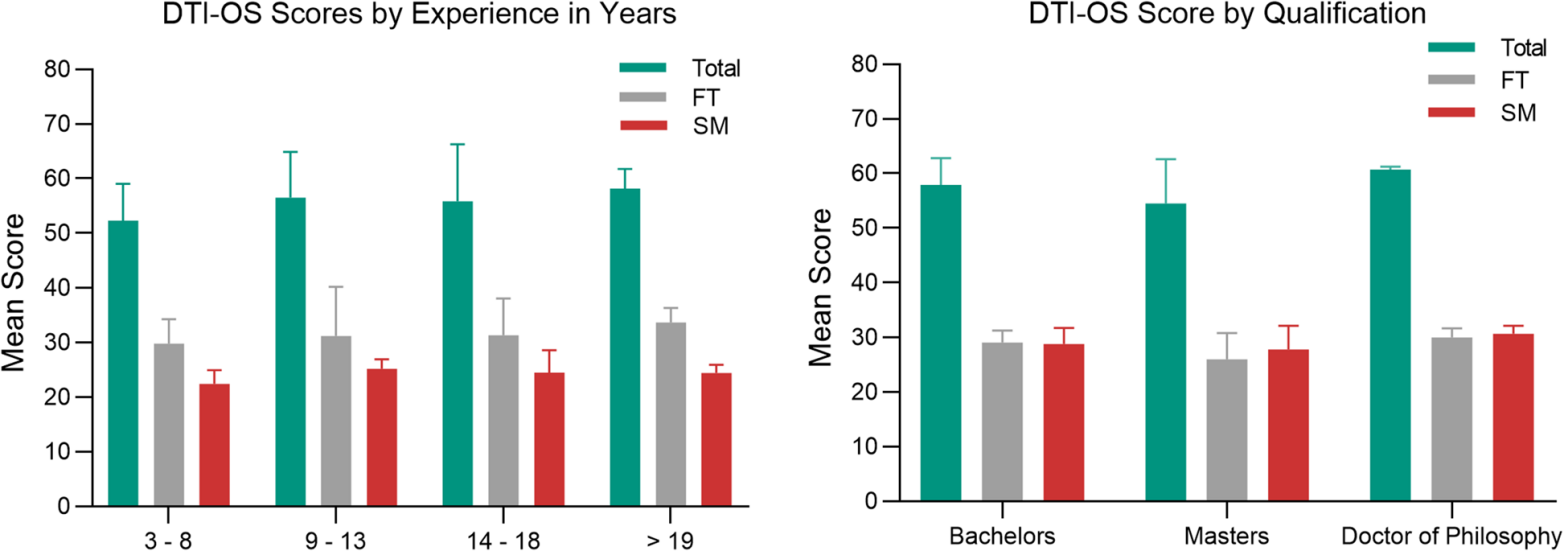
The mean total score and subdomains for the DTI-OS by clinical experience and qualification. **A** clinical experience of participants measured in years from the expert group; **B** qualification of participants in the expert group

Factors such as clinical experience measured in years of clinical practice and age (Pearson’s correlation *r* = .54, *P* = <.001 and *r* = .44 *P* = .01 respectively) showed a statistically significant positive relationship with the DTI-OS total scores and subdomains (see Fig. 6A). A post hoc comparison using Tukey HSD test indicated that the greatest statistical difference was between 3 and 8 years’ experience and 14–18 years’ experience. In contrast there was no statistically significant relationship between scores and level of qualification (Pearson’s correlation *r* = − .16, *P* = .74) shown in Fig. 6B.

### Reliability

The overall classical test theory reliability for internal consistency was α = 0.92 for the DTI-OS total scores, α = 0.90 (k = 6) for flexibility in thinking and α = 0.83 (k = 6) for structured memory.

External reliability was determined by analyzing the DTI-OS scores from attempts 1 and 2 by the expert group. Using participants responses for both attempts the ICC and their 95% confidence intervals were calculated using an absolute agreement, 1-way random effects model [32]. These and mean scores of both attempts are in Table 5 and show excellent reliability for the DTI-OS.

**Table 5:** External reliability of the DTI-OS

Score	Maximum possible value	Means (± SDs)		ICC average (upper and lower bounds, 95% CI)
		First administration Experts (***n*** = 32)	Second administration Experts (***n*** = 27)	
DTI-OS Total	72	55.87 (**±** 6.89)	56.22 (**±** 6.81)	0.922 (0.83–0.96)*
DTI-OS SM	36	28.43 (± 3.67)	28.66 (**±** 4.06)	0.90 (0.79–0.96)*
DTI-OS FT	36	27.44 (± 4.14)	27.85 (**±** 3.51)	0.82 (0.61–0.92)*
DTI-O Total	246	182.71 (**±**19.84)	183.24 (±18.49)	0.93 (0.85–0.97)
DTI-O SM	120	74.71 (±6.90)	71.04 (±8.05)	0.87 (0.59–0.95)
DTI-O FT	126	70.05 (±10.18)	73.31 (±9.55)	0.84 (0.34–0.95)

*Significant at *P* < .001

Abbreviations: *DTI-O* Diagnostic Thinking Inventory for Optometry, *DTI-OS* Diagnostic Thinking Inventory for Optometry Short, *FT* flexibility in thinking, *SM* structured memory

#### Rasch analysis – reliability

The person separation index for the total 12-item DTI-OS (0.90), the six-item SM subdomain (0.84), and the six-item FT subdomain (0.77) all showed that the survey had good internal reliability. As previously mentioned, the data from the repeat completions of the survey by the experts at time points 1 and 2 were included in the Rasch analysis by stacking the data [24]. The results from analyzing the expert data alone (χ^2^ = 51.94, df = 24, *P* = 0.0008; item (0.08 ± 1.06) and person (0.21 ± 1.01) fit residuals; person separation index = 0.84) were similar to the results from the pooled expert and novice data. The person ability estimates at time 1 (1.55 ± 1.06 logits) and time 2 (1.48 ± 0.98 logits) were not significantly different from each other (one-way ANOVA, F = 0.07, *P* = 0.80), and the mean difference in individual ability estimates between time 1 and time 2 was 0.08 ± 0.65 logits (median = 0; range: − 1.71 to 1.93 logits).

## Discussion

The objective of this study was to test the validity and reliability of the DTI-O for use as a self-reflective tool to measure diagnostic reasoning ability in trainee and experienced optometrists and to investigate the underlying structure of a relatively large item number inventory to form a shorter, potentially more efficient version through item reduction and validate this tool [31]. The face validity of the DTI-O was confirmed by a panel of experts who reviewed the 41-items and concluded they were assessing diagnostic reasoning and that the subdomains were relevant to the context of optometry. The construct validity was determined with Rasch measurement theory and Exploratory Factor Analysis and these found that experts scored higher than novices.

Rasch analysis determined that there is multidimensionality of the DTI-O and Exploratory Factor analysis through item reduction showed that the DTI-OS and the two defined subdomains were measuring a single factor; diagnostic reasoning. The fewest number of questions needed to measure and understand this factor were identified, resulting in 12-items (evenly split between structured knowledge and flexibility in thinking domains) that accounted for the majority of variance of the original inventory, and these were used in further validity and reliability analysis of the DTI-OS (Additional file 2). The results of an independent *t*-test between the two groups demonstrated the construct validity of the shortened inventory with the expert group scoring higher than the novice group (see Table 4).

A test-retest method of administrating the DTI-O at two different time points proved external reliability using ICC analysis and stacked Rasch analysis. The DTI-O instructions asked participants to reflect on their own diagnostic reasoning in relation to clinical experiences and select their response to reflect where they felt their diagnostic reasoning is most of the time. This potentially allowed for recent clinical exposure to skew the results, however the ICC analysis confirmed there were no patterns for variance in responses from attempts 1 and 2 in the expert group. This analysis did show that on repeated administration the DTI-OS produces statistically similar total scores and scores for both subdomains (Table 5). The Rasch analysis with the DTI-OS 12-item survey indicate that this tool can facilitate tracking of skill development in a novice population over time given the repeatability of the survey in the expert sample. This supports the hypothesis that the results of the DTI-OS are reliable.

The internal consistency of the DTI-OS further supports the reliability of the inventory. The Cronbach’s alpha values indicate excellent internal reliability for the total score (α = 0.92) indicating the inventory measures the same thing, and subdomains of flexibility in thinking (α = 0.83) and structured memory (α = 0.90). The classical test theory reliability indices were similar to the person separation indices determined through Rasch analysis (0.90, 0.77, and 0.84 respectively), confirming excellent internal reliability. These values are slightly higher than the original DTI validation study (α = 0.83 for the total score, α = 0.72 for flexibility in thinking and α = 0.74 for structured knowledge) [7]. This supports the use of the DTI-OS as a valid tool that reliably measures self-reported diagnostic reasoning in optometry and that it could be used in future studies as a means to explore diagnostic reasoning in optometry.

The validity and reliability investigations in this study determined that the DTI-O and DTI-OS are valid and reliable self-reflective inventories to measure diagnostic reasoning in optometry, both for trainee students and practicing clinicians. The face validity of the DTI-O was established by a panel of experts and the mean scores in Table 4 are comparable to those reported by Bordage et al., [7] For example the mean total DTI scores for third-year and first-year medical students were 158.3 (**±**18.5) and 153.9 (**±** 18.2) respectively, compared to the mean score of 155.62 (**±** 12.70) for second-year optometry students with the DTI-O. Further investigation with a larger sample size will enable definition of standard scores for the DTI-O. Similar to Bordage et al.*,* the mean total scores increase with clinical experience, until Registrar level (medical) or 8–14 years (optometry) and then decline as areas of expertise begin to develop.

In this study optometrists achieved higher total scores with increasing clinical experience (see Fig. 5). This indicates that the DTI-OS may be used in investigating learning characteristics, evaluating interventions and developing curriculum [33, 34]. For example flexibility in thinking increased with higher levels of clinical experience and implies optometrists with more years’ experience self-report they are more able to use multiple methods of investigation and manage conflicting information. Since entry level qualifications for optometry have risen in the past decade, when analysing the responses by qualification, most participants with a Master’s level of qualification had fewer years of clinical experience than those with a Bachelors qualification. This explains why practising optometrists with a Master’s degree scored lower than those with a Bachelor’s degree. Allowing for this fact, the results suggest the DTI-OS could be used in optometry education and optometry practice to evaluate diagnostic reasoning in relation to clinical experience and for continuing professional development. It is vital that optometrists maintain competency in all areas, regardless of areas of clinical expertise or special interests. Due to the context-neutral design, the DTIO-S can be applied to different areas of practice and could be used to assist qualified optometrists in targeting their professional development activities to areas of greatest need. Indeed, changes to continuing professional education requirements in several jurisdictions require optometrists to actively engage in critical reflection of their practice strengths and weaknesses and develop learning plans. Utilization of the DTI=OS, as part of a self-reflective process, would enable quantification of a practitioner’s self-evaluation of their overall diagnostic reasoning skills, poviding insights as to whether further learning should be targeted towards knowledge acquisition or flexible application.

In an optometric education setting, the DTI-OS could be used as a scaffold to support diagnostic reasoning development in optometric education. When students are given specific training and guidance on diagnostic reasoning, research demonstrates that this skill can improve [1]. The challenge in optometric education is that diagnostic reasoning is an essential skill for students to gain but a difficult skill to teach or implement in a curriculum when the diagnostic reasoning process in optometry practice is not clearly understood. Additionally identifying deficiencies in diagnostic reasoning is important as it has been shown to be related to clinical performance [35]. The DTI-OS, as a valid and reliable self-reflective inventory, could be used to facilitate and enhance the teaching of diagnostic reasoning and the assessment of this skill.

## Limitations

Firstly, our sample size is small for factor analysis and is limited by the cohort size of second-year students. The minimum number of participants is debated across the literature and our number of participants can be supported by the high Kaiser-Myer-Olkin value and commonalities for all items. Secondly, our sample of students do not represent the population across institutions. As the inventory has been validated and its reliability proven, our future investigations seek to generate standard scores by incorporating a larger sample size and multiple institutions. This will be made achievable with the DTI-O and DTI-OS being released as an online inventory (Additional file 1).

Thirdly, the DTI-O is designed as a self-assessment of diagnostic reasoning ability and potential inherent limitations could exist when interpreting results. One study on using the original DTI reported it is influenced by cognitive and noncognitive factors [34]. Factors that might influence the DTI-O scores are reflective ability, self-confidence, self-criticism, cognitive bias, motivation and experience. It should be considered in future evaluation studies that these factors were not investigated in this validation study. Despite these limitations, the study highlights the potential for future use of these self-reflective diagnostic reasoning inventories.

## Conclusions

In conclusion the DTI-O is a valid self-assessment tool to measure diagnostic reasoning in optometry and in the two domains of flexibility in thinking and structured memory. The DTI-OS offers a shorter version of this tool, which may make it more amendable to use as a longitudinal tracking tool for diagnostic reasoning. Further investigations have the potential to generate standard scores to enable a basis for comparative studies. As the scope of optometry practice is ever-evolving it is important for practitioners to reflect on their competence in diagnostic reasoning in new contexts and areas of practice. As a subjective measure, independent of knowledge, these inventories have potential use as evaluation tools in optometry. At a university level they may provide a measure of diagnostic reasoning in relation to learning characteristics, intervention evaluation and curriculum development.

## Supplementary Information

**Additional file 1.** Diagnostic Thinking Inventory for Optometry (DTI-O). A 41-item inventory to self-assess clinical reasoning skills in optometry students and practitioners.

**Additional file 2.** Diagnostic Thinking Inventory for Optometry Short (DTI-OS). A 12-item inventory to self-assess clinical reasoning in optometry students and practitioners.

## Data Availability

The dataset generated and analyzed during the current study is available to the authors but is not publicly available due to ethical guidelines. The datasets used and/or analyzed during the current study available from the corresponding author on reasonable request.

## Abbreviations

DTI: Diagnostic Thinking Inventory
DTI-O: Diagnostic Thinking Inventory for Optometry
FT: Flexibility in thinking
SM: Structured memory
DTI-OS: Diagnostic Thinking Inventory for Optometry Short
±SDs: ±Standard Deviations

## References

[1] Facione N, Facione P (2008). Critical thinking and clinical judgment. J Optometric Ed.

[2] Kiely PM, Slater J (2015). Optometry Australia entry-level competency standards for optometry 2014. Clin Exp Optom.

[3] Ende J, American College of Physicians. Theory and practice of teaching medicine. Philadelphia: American College of Physicians; 2010. xxv, p. 161.

[4] Global Competency-Based Model. World Council of Optometry; 2015 2005.

[5] Faucher C, Tardif J, Chamberland M (2012). Optometrists' clinical reasoning made explicit: a qualitative study. Optom Vis Sci.

[6] Marcum JA (2012). An integrated model of clinical reasoning: dual-process theory of cognition and metacognition. J Eval Clin Pract.

[7] Bordage G, Grant J, Marsden P (1990). Quantitative assessment of diagnostic ability. Med Educ.

[8] Beullens J, Struyf E, Van Damme B (2006). Diagnostic ability in relation to clinical seminars and extended-matching questions examinations. Med Educ.

[9] Stieger S, Praschinger A, Kletter K, Kainberger F (2011). Diagnostic grand rounds: a new teaching concept to train diagnostic reasoning. Eur J Radiol.

[10] Jerant A. Validity of Scores. 2004.

[11] Windish DM, Price EG, Clever SL, Magaziner JL, Thomas PA (2005). Teaching medical students the important connection between communication and clinical reasoning. J Gen Intern Med.

[12] Findyartini A, Hawthorne L, McColl G, Chiavaroli N (2016). How clinical reasoning is taught and learned: cultural perspectives from the University of Melbourne and Universitas Indonesia. BMC Med Educ.

[13] Heinerichs S, Vela LI, Drouin JM (2013). A learner-centered technique and clinical reasoning, reflection, and case presentation attributes in athletic training students. J Ath Train.

[14] Jones UF (2009). The reliability and validity of the Bordage, Grant & Marsden diagnostic thinking inventory for use with physiotherapists. Medl Teach.

[15] Kicklighter T, Barnum M, Geisler PR, Martin M (2016). Validation of the quantitative diagnostic thinking inventory for athletic training: a pilot study. Athl Train Educ J.

[16] Kane MT (2016). Explicating validity. Assess Educ Princ Policy Pract.

[17] World Medical A (2013). World medical association declaration of Helsinki: ethical principles for medical research involving human subjects. JAMA.

[18] De Vaus DA. Surveys in social research. Sixth edition. ed. Abingdon, Oxon: Routledge; 2014. xviii, p. 382.

[19] Andrich D (1978). A rating formulation for ordered response categories. Psychometrika.

[20] Salkind NJ (2010). Encyclopedia of research design (Vols. 1-0).

[21] Andrich D, Sheridan BS, Luo G (2015). RUMM 2030: Rasch unidimensional measurement models.

[22] Andrich D, Marais I. Violations of the Assumption of Independence II—The Polytomous Rasch Model. In: A Course in Rasch Measurement Theory. Springer Texts in Education. Singapore: Springer; 2019. 10.1007/978-981-13-7496-8_24.

[23] Tennant A, Conaghan PG (2007). The Rasch measurement model in rheumatology: what is it and why use it? When should it be applied, and what should one look for in a Rasch paper?. Arthritis Rheuma.

[24] Munro BH (2005). Statistical methods for health care research.

[25] Hayes SC (2002). Acceptance, Mindfulness, and science. Clin Psychol Sci Pract.

[26] Tabachnick BG, Fidell LS, Ullman JB (2007). Using multivariate statistics.

[27] Anna BC, Jason O (2005). Best practices in exploratory factor analysis: four recommendations for getting the most from your analysis. Pract Assess Res Eval.

[28] Wright BD. Rack and Stack: Time 1 vs. time 2. Rasch measurement Transactions. 2003;17(1). http://www.rasch.org/rmt/rmt171a.htm.

[29] Fisher WP Jr. Rating scale instrument quality criteria. Rasch Measure Transact. 2007;21:1095. http://www.rasch.org/rmt/rmt211a.htm.

[30] Linacre JM, Tennant A (2009). More about critical eigenvalue sizes (variances) in standardized-residual principal components analysis (PCA). Rasch Measure Transact.

[31] Andrich D, Humphry S, Marais I (2012). Quantifying local, response dependence between two Polytomous items using the Rasch model. Appl Psychol Meas.

[32] Koo TK, Li MY (2016). A guideline of selecting and reporting Intraclass correlation coefficients for reliability research. J Chiropr Med.

[33] Sobral DT (1995). Diagnostic ability of medical students in relation to their learning characteristics and preclinical background. Med Educ.

[34] Round AP (1999). Teaching clinical reasoning--a preliminary controlled study. Med Educ.

[35] Denial A (2008). Association of Critical Thinking Skills with Clinical Performance in fourth-year optometry students. J Optometric Ed.

