# Supplementary material for: A cohort study for the development and validation of a reflective inventory to quantify diagnostic reasoning skills in optometry practice
Source: BMC Med Educ. 2022 Jul 11;22:536. doi: 10.1186/s12909-022-03493-6 (PMC9277884; doi:10.1186/s12909-022-03493-6)
Supplement: Supplementary file 1 — Additional file 1. Diagnostic Thinking Inventory for Optometry (DTI-O). A 41-item inventory to self-assess clinical reasoning skills in optometry students and practitioners. [file 12909_2022_3493_MOESM1_ESM.pdf]

Diagnostic Thinking Survey

Completion of this survey indicates consent

Participant Name:…………………………………..

Student ID Number (if applicable):…………………………….…….………

What gender are you? (Please circle)

- Male
- Female
- Other
- Prefer not to say

What age group are you? (please circle)

- 18 to 21
- 22 to 30
- 30 to 40
- 40 to 50
- 50 +

Please specify what level of qualification are you? *(i.e. what unit are you currently enrolled in, for example HMO 303/304, in your course or what year did you graduated with a qualification in optometry?)*

…………………………………………………..

Are you currently studying Optometry? *If yes please answer the following questions, if no please commence the survey.*

Have you previously studied Optometry or another healthcare field at another university? (Please circle) Yes / No

Have you previously studied another course in higher education? (Please circle) Yes / No

If you answered yes to either of the above question please specify:……………………………………………………………………………

Have you previously been enrolled in your current unit (HMO203, HMO204 HMO303, HMO304, HMO305 or HMO306) prior to this academic calendar year? Yes / No

INSTRUCTIONS: This inventory contains 41 items concerning your diagnostic thinking. Each item contains a stem, two accompanying statements and a rating scale. The scale refers to the continuum between two statements. Please put a cross (X) in the space between the markers which best describes your position on the continuum.

Do not try to work out any underlying meaning to each item; there is no right or wrong answer. Only the sum of the items will have a significance. Simply respond as spontaneously as you can by indicating how you actually diagnose and not how you think you should (even for those with little clinical experience). You will often find that you actual do things associate with both statements for a given item; your cross will indicate which one you do most often. Do not put your mark on the mid marker; if you hesitate between two statements, please decide which one reflects what you do most often. You may think there are other alternatives besides the two statements given (and there can be more than two in many instances), please make a choice on the basis of the two statements provided. It will take you about 15 to 20 minutes to complete this inventory.

If you are a student who has currently had no clinical exposure to patients please reflect on your experience in your studies, for example problem-based learning (PBL) and team-based learning (TBL) opportunities.

---

1) When the patient presents their symptoms,

I think of their symptoms in the precise words used by the patient	I think of the symptoms in more abstract terms than the expressions actually used (e.g. ‘4-day duration’ becomes ‘acute’; ‘two-eyes becomes ‘bilateral’)

2) In considering each diagnosis,

I try to evaluate their relative importance (in terms of impact for the patient)	I try to give them equal importance or weighting

3) In thinking of diagnostic possibilities,

I think of diagnostic possibilities early on in the case	First I collect the clinical information and then think about it

4) When I am questioning a patient about their history,

I often seem to get one idea stuck in my mind about what might be wrong	I find it easy to explore various possible differential diagnoses

5) Through history taking,

If I follow the patient’s line of thought I tend to lose my own thread	I can still keep my own ideas clear even if I follow the patient’s line of thought

6) When it comes to making up my mind about a diagnosis,

I do not mind postponing my diagnostic decisions about a case	└─┴─┴─┴─┴─┴─┘	I feel obligated to go for one diagnosis or another even if I am not very certain

7) Once the patient has clearly presented their symptoms and signs,

I think about them in my mind in the patient's own words	└─┴─┴─┴─┴─┴─┘	I translate them in my mind into medical terms (e.g. "eye-strain" becomes "asthenopia", "flashes" become "photopsia")

8) In relation to the routine history,

I often feel that I did not sufficiently cover the routine history	└─┴─┴─┴─┴─┴─┘	I usually cover the routine history to my satisfaction

9) As the patient tells their story and the case history unfolds,

I often find it difficult to remember what has been said	└─┴─┴─┴─┴─┴─┘	I can usually keep track in my mind of what has been said

10) During the case history, I find that,

Some key pieces of information seem to leap out at me	└─┴─┴─┴─┴─┴─┘	New information does not often make me have more ideas

11) When I cannot make sense of a patient's symptoms,

I move on and gather new information to trigger new ideas	└─┴─┴─┴─┴─┴─┘	I ask the patient to define those symptoms more clearly

12) In considering diagnostic possibilities,

I often come up with unlikely diagnoses	└─┴─┴─┴─┴─┴─┘	I am usually in the right area

13) While I am collecting information about a patient,

The various items of information usually seem to group themselves together in my mind	└─┴─┴─┴─┴─┴─┘	I often have difficulty seeing how the pieces of information relate to each other

14) When the diagnosis becomes known and I realise that I have missed it initially,

It is often because I knew the disease but failed to think about it	└─┴─┴─┴─┴─┴─┘	It is often because I did not know enough about the disease

15) During the case history,

I cannot bring myself to dismiss some information as irrelevant	└─┴─┴─┴─┴─┴─┘	I am quite happy to dismiss some information as irrelevant

16) When I cannot make sense of the patient's symptoms and signs,

I move on to get new information and a new perspective	└─┴─┴─┴─┴─┴─┘	I look at them from a difference perspective before moving on

17) When considering a number of diagnoses,

The diagnoses tend to be related to one another	└─┴─┴─┴─┴─┴─┘	The diagnoses tend to be scattered

18) When a possible diagnosis comes to mind,

I usually find myself anticipating possible abnormal signs and symptoms that go with that diagnosis	└─┴─┴─┴─┴─┴─┘	Quite often, it does not help me to decide what to ask the patient

19) When I know very little about a particular type of disease,

I can still usually come up with a diagnosis	└─┴─┴─┴─┴─┴─┘	I have great difficulty reaching a diagnosis

20) In considering the patient's signs and symptoms,

I think of them in absolute terms as stated by the patient	└─┴─┴─┴─┴─┴─┘	I think of them in terms of possible opposites (e.g. progressive vs sudden; unilateral vs bilateral)

21) When I know a lot about a particular disease and have to make a diagnosis,

I find it relatively easy to pin down a diagnosis	└─┴─┴─┴─┴─┴─┘	I often seem to be all over the place and have difficulty pinning down a diagnosis

22) As the history progresses and I already have some ideas about the possible diagnosis(es),

New information makes me have more ideas	New information does not often make me have more ideas

23) When I am taking a history, I find that,

I can get new ideas just by going over the existing information in my mind	I need to have new information to make me have a new idea about the case

24) When the patient uses imprecise or ambiguous expressions

I let him or her go on to maintain the flow of the interview	I make him or her clarify precisely what he or she means before going on

25) After a case history with a patient,

I rarely think of other things that I should have asked in relation to the patient's disorder	I often think of other things that I should have asked in relation to the patient's disorder

26) When a piece of information comes along and makes me think of a possible diagnosis,

It often makes me go back to previous information to see if things fit together or not	It rarely makes me review the information that I gathered previously

27) In relation to the diagnosis I eventually make,

I usually have very few doubts	I often feel too uncertain for my own comfort

28) In making a diagnostic decision,

I decide by considering each possible diagnosis separately on its own merits	I decide by comparing and contrasting the various possible diagnoses

29) When I know a lot about a particular type of disease and have to make a diagnosis,

I check up on most possibilities before reaching a decision	I often have lots of ideas that I don't explore further

30) As the case unfolds,

I do not find it useful to summarise as I go on	└─┴─┴─┴─┴─┴─┘	I periodically take stock of the data and my ideas

31) When I reach my diagnostic decisions,

There is often left-over information I have just forgotten about	└─┴─┴─┴─┴─┴─┘	I usually will have considered all the information.

32) When I have got an idea about what might be wrong with the patient

I feel most comfortable if I can follow it up without being diverted	└─┴─┴─┴─┴─┴─┘	I feel happy to go off on another track and come back to my original ideas later

33) When I come up with a broad idea as to what might be wrong with the patient,

I can usually proceed to a specific diagnosis	└─┴─┴─┴─┴─┴─┘	I find it difficult to put it into specific terms

34) Throughout acquiring a case history,

I manage to test my ideas even if I let the patient control the interview	└─┴─┴─┴─┴─┴─┘	I am only successful if I can control the direction of the interview

35) In relation to choosing from among the diagnostic ideas that I have,

I am usually not capable of wholly ruling out any of the ideas I have had	└─┴─┴─┴─┴─┴─┘	I am capable of ruling out most of my ideas completely

36) Once I have made up my mind about a patient,

I am prepared to change my mind	└─┴─┴─┴─┴─┴─┘	I really do not like to change my mind

37) When I consider my diagnostic ideas, I do so on the basis of,

The case as a whole so far	└─┴─┴─┴─┴─┴─┘	A few outstanding symptoms or signs

38) If I do not know what to make of a clinical interview

I can readily see the information in new ways	I find it always difficult to see the information in new ways

39) When I do further diagnostic tests,

I do it as part of the routine clinical investigation	I do it expecting specific information or supporting evidence

40) In considering differential diagnostic possibilities,

I compare and contrast the possible diagnoses	I consider each diagnosis separately on its own merits

41) In terms of a way I take case history,

I usually cover the ground that I need to during the interview	Quite often I do not ask all the questions that I should at the time
