# Supplementary material for: A cohort study for the development and validation of a reflective inventory to quantify diagnostic reasoning skills in optometry practice
Source: BMC Med Educ. 2022 Jul 11;22:536. doi: 10.1186/s12909-022-03493-6 (PMC9277884; doi:10.1186/s12909-022-03493-6)
Supplement: Supplementary file 2 — Additional file 2. Diagnostic Thinking Inventory for Optometry Short (DTI-OS). A 12-item inventory to self-assess clinical reasoning in optometry students and practitioners. [file 12909_2022_3493_MOESM2_ESM.pdf]

DEAKIN UNIVERSITY

Diagnostic Thinking Survey

INSTRUCTIONS: This inventory contains 12 items concerning your diagnostic thinking. Each item contains a stem, two accompanying statements and a rating scale. The scale refers to the continuum between two statements. Please put a cross (X) in the space between the markers which best describes your position on the continuum.

Do not try to work out any underlying meaning to each item; there is no right or wrong answer. Only the sum of the items will have a significance. Simply respond as spontaneously as you can by indicating how you actually diagnose and not how you think you should (even for those with little clinical experience). You will often find that you actual do things associate with both statements for a given item; your cross will indicate which one you do most often. Do not put your mark on the mid marker; if you hesitate between two statements, please decide which one reflects what you do most often. You may think there are other alternatives besides the two statements given (and there can be more than two in many instances), please make a choice on the basis of the two statements provided. It will take you about 15 to 20 minutes to complete this inventory.

If you are a student who has currently had no clinical exposure to patients please reflect on your experience in your studies, for example problem-based learning (PBL) and team-based learning (TBL) opportunities.

1) When I am questioning a patient about their history,

I often seem to get one idea stuck in my mind about what might be wrong

I find it easy to explore various possible differential diagnoses

2) Through history taking,

If I follow the patient’s line of thought I tend to lose my own thread

I can still keep my own ideas clear even if I follow the patient’s line of thought

3) In relation to the routine history,

I often feel that I did not sufficiently cover the routine history

I usually cover the routine history to my satisfaction

4) As the patient tells their story and the case history unfolds,

I often find it difficult to remember what has been said

I can usually keep track in my mind of what has been said

5) While I am collecting information about a patient,

The various items of information usually seem to group themselves together in my mind	I often have difficulty seeing how the pieces of information relate to each other

6) During the case history,

I cannot bring myself to dismiss some information as irrelevant	I am quite happy to dismiss some information as irrelevant

7) When I know very little about a particular type of disease,

I can still usually come up with a diagnosis	I have great difficulty reaching a diagnosis

8) In relation to the diagnosis I eventually make,

I usually have very few doubts	I often feel too uncertain for my own comfort

9) When I reach my diagnostic decisions,

There is often left-over information I have just forgotten about	I usually will have considered all the information.

10) When I come up with a broad idea as to what might be wrong with the patient,

I can usually proceed to a specific diagnosis

I find it difficult to put it into specific terms

11) If I do not know what to make of a clinical interview,

I can readily see the information in new ways

I find it always difficult to see the information in new ways

12) In terms of a way I take case history,

I usually cover the ground that I need to during the interview

Quite often I do not ask all the questions that I should at the time
